# The Effect of a Variable Disc Pad Friction Coefficient for the Mechanical Brake System of a Railway Vehicle

**DOI:** 10.1371/journal.pone.0135459

**Published:** 2015-08-12

**Authors:** Nam-Jin Lee, Chul-Goo Kang

**Affiliations:** 1 Bogie Development Team, Hyundai-Rotem Company, Uiwang-shi, Gyeonggi-do, South Korea; 2 Department of Mechanical Engineering, Konkuk University, Gwangjin-gu, Seoul, South Korea; Massachusetts Institute Of Technology, UNITED STATES

## Abstract

A brake hardware-in-the-loop simulation (HILS) system for a railway vehicle is widely applied to estimate and validate braking performance in research studies and field tests. When we develop a simulation model for a full vehicle system, the characteristics of all components are generally properly simplified based on the understanding of each component’s purpose and interaction with other components. The friction coefficient between the brake disc and the pad used in simulations has been conventionally considered constant, and the effect of a variable friction coefficient is ignored with the assumption that the variability affects the performance of the vehicle braking very little. However, the friction coefficient of a disc pad changes significantly within a range due to environmental conditions, and thus, the friction coefficient can affect the performance of the brakes considerably, especially on the wheel slide. In this paper, we apply a variable friction coefficient and analyzed the effects of the variable friction coefficient on a mechanical brake system of a railway vehicle. We introduce a mathematical formula for the variable friction coefficient in which the variable friction is represented by two variables and five parameters. The proposed formula is applied to real-time simulations using a brake HILS system, and the effectiveness of the formula is verified experimentally by testing the mechanical braking performance of the brake HILS system.

## Introduction

It is difficult and complicated to estimate the performance of the brake system of a railway vehicle on vehicle running conditions because it is dangerous to test various emergency situations. The brake system is composed of many components, such as electronic control units, pneumatic operating units, mechanical brake actuators, and friction materials, and they interact with each other dynamically [1,2]. A hardware-in-the-loop simulation (HILS) of a railway vehicle is considered to be one solution for reducing the risks and costs of field tests of a running railway vehicle. Recently, diverse applications of the HILS system have been reported, especially in the area of vehicle brake systems [3–6].

The brake HILS system is generally composed of two separate parts. One part comprises the simulation software tools used to implement the mathematical models of running car dynamics in real-time, and the other part comprises the actual hardware of the brake unit used in an actual railway vehicle. The interface between the two parts should provide environments similar to the actual connections that exist in the railway vehicle.

The performance of the brake HILS system depends on the accuracy of the mathematical models of real-time simulations as well as the accuracy of the real-time implementation (e.g., latency). When we develop a simulation model for a full vehicle system, the characteristics of all components are generally simplified properly based on the understanding of each component’s purpose and its interaction with other components. The friction coefficient between a brake disc and a pad is an ambiguous characteristic to model. The friction coefficient between the brake disc and the pad in simulations is usually considered to be a constant and is referred to as the average friction coefficient. The effect of a variable friction coefficient is ignored with the assumption that it affects the performance of the vehicle braking very little. However, the friction coefficient changes significantly over a range due to environmental conditions, and thus, it can affect braking performance considerably, especially on the wheel slide.

Previously, the friction coefficient between a disc and a pad was studied in the context of how to measure or control the quality of the friction materials [7,8]. The local tribological behavior has been studied using reduced scale tribometer testing [9], coupling between the friction mechanisms and thermal phenomena at the pad–disc contact [10,11], and the pressure effect on the friction characteristics [12]. Parallel to tribological studies, systemic approaches to full brake systems have been conducted. A HILS system of a railway vehicle or a road car has been proposed and studied for braking and wheel slide protection [13–15] and for active control to improve performance [16,17]. To simulate the drive dynamics, which include the brake system, experimental and theoretical approaches to modeling wheel-rail contact have been developed [18–20]. The variable friction coefficient of a brake pad has been used to estimate the adhesion force in the reference [21], but this study did not consider the effect of temperature on the coefficient of friction.

In this paper, the variation of the friction coefficient between a disc and a pad is taken into account when analyzing the brake performance of a railway vehicle. A mathematical formula describing the friction characteristics is proposed and includes a thermal effect, which is able to represent a general attribute of variable friction using two variables and five parameters derived from the dynamometer tests of specific discs and pads. Then, the effect of the variable friction on the braking performance is demonstrated using the brake HILS system developed in our laboratory.

In section 2, a formula for the variable friction coefficient is proposed, and in section 3, a mathematical model of a vehicle including the wheel-rail contact and brake devices is presented. In section 4, we describe the brake HILS system developed in our laboratory, and we demonstrate the validity of the proposed variable friction coefficient through experimental tests using the HILS system in section 5. Section 6 summarizes and concludes the work.

## Variable Friction Coefficient

**Fig 1:**
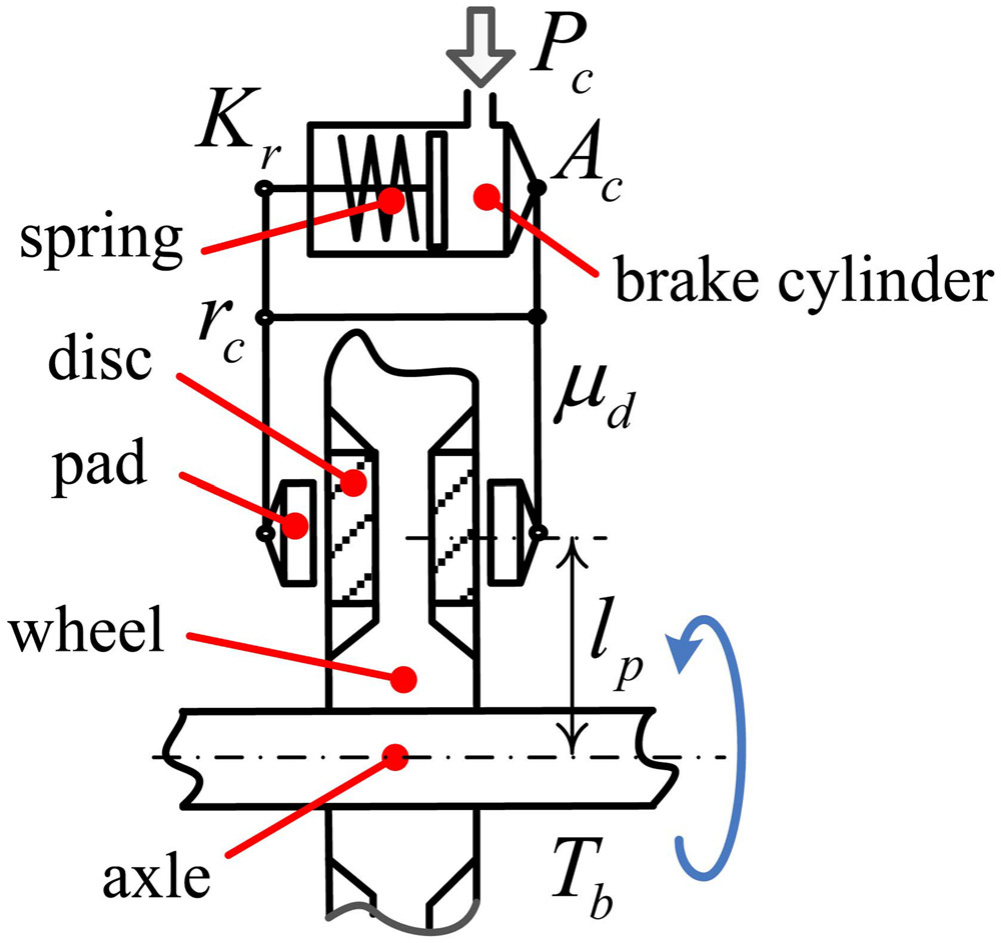
Schematic of the wheel-mounted disc and brake caliper with pads. The braking force is generated by squeezing the wheel using two pads. The braking force of the pad is controlled by the pneumatic pressure *P*_*c*_ of the brake cylinder via a lever mechanism with a pivot at *r*_*c*_. The braking force is released by the spring installed inside the brake cylinder.

A mechanical brake system of a railway vehicle primarily converts kinetic energy of the vehicle to heat energy through friction force. Mechanical braking is a conventional and reliable method to stop the railway vehicle compared with electric braking. The target vehicle in this research is a railway vehicle that is in service in Korea and has a wheel-mounted disc brake. The friction force in the wheel-mounted disc brake is generated by relative movement between the friction materials via a normal force that is controlled by the pneumatic actuators and valves. Fig 1 shows a schematic of the wheel-mounted disc and the brake caliper with pads [22].

The braking torque of one wheelset is controlled by air pressure coming from the BOU (brake operating unit), and the braking torque is given by [22]

$$T_{b}(t)=\mu_{d}\;(t)\left(A_{c}\;P_{c}(t)-K_{r}\right)\;r_{c}\;l_{p}\;N_{d}$$ (1)

where *T*_*b*_ is the brake torque acting on a wheelset, *A*_*c*_ is the effective piston area, *P*_*c*_ is the air pressure controlled by the BOU, *K*_*r*_ is the retaining spring force of the piston in the cylinder, *r*_*c*_ is the lever ratio of the caliper, *l*_*p*_ is the radius of the effective braking force, and *N*_*d*_ is the number of wheel discs. *μ*_*d*_(*t*) represents the variable friction coefficient between the disc and pad.

The characteristics of the contact between the brake disc and pad is generally measured through dynamometer tests using the methods in references [7,8], and then the obtained *average* friction coefficient is used for the brake control and the brake performance calculations. However, the measured instantaneous friction coefficient changes within the specified range during a braking period. This variation of the friction coefficient affects the short-term brake behaviors, such as the jerk of the vehicle or the slip-slides of the wheel, even though the average friction coefficient is sufficient to estimate the brake performance under normal running conditions.

The tribological phenomena for a disc-pad pair includes the heat transfer, which is complex and time-consuming to compute accurately using a theoretical mathematical model, so it is not appropriate to include a heat transfer model in real-time simulations when using the HILS system. In this study, alternately, we focused on how to represent the instantaneous friction coefficients between the disc and the pad in an equation form that included the thermal effects in the HILS system of the railway vehicle.

The friction coefficient is normally expected to primarily depend on the temperature of the surfaces in contact and the relative speed of the contacting surfaces. The pattern of the instantaneous friction coefficient at the beginning of braking or at low speed is observed to be relatively high compared with the steady-state value. With this phenomenal understanding based on observations, Eq (2) is introduced in this paper to represent the characteristics of varying friction between the brake disc and the pad.

μd(t)=μd0 (nv e−m vvd(t)+1) (nT e−m TTd(t)+1) (2)

where *μ*_*d0*_ is the steady-state friction coefficient between the disc and the pad, *n*_*v*_ is a multiplication factor that is caused by the friction speed, *m*_*v*_ is a parametric coefficient of the exponential function of the friction speed, *n*_*T*_ is a multiplication factor caused by the increase in temperature, *m*_*T*_ is a parametric coefficient of the exponential function of the increase in the temperature, *c* is a correction constant. The friction speed *v*_*d*_ is the relative speed between the disc and the pad in m/s, and *T*_*d*_ is the increase in temperature of the disc surface in Celsius. In Eq (2), the variable friction coefficient *μ*_*d*_ is a function of the two variables *v*_*d*_ and *T*_*d*,_ and the five parameters *μ*_*d0*_, *n*_*v*_, *n*_*T*_, *m*_*v*_, and *m*_*T*_.

The proposed equation (Eq (2)) for a variable friction coefficient considers the effect of the increase in temperature of the friction materials and the friction speed. More specifically, Eq (2) is composed of the multiplication of three parts: the first part is the steady-state value of the friction coefficient, the second part is a correction due to the friction speed, and the third part is a correction due to the increase in temperature of the friction materials.

**Fig 2:**
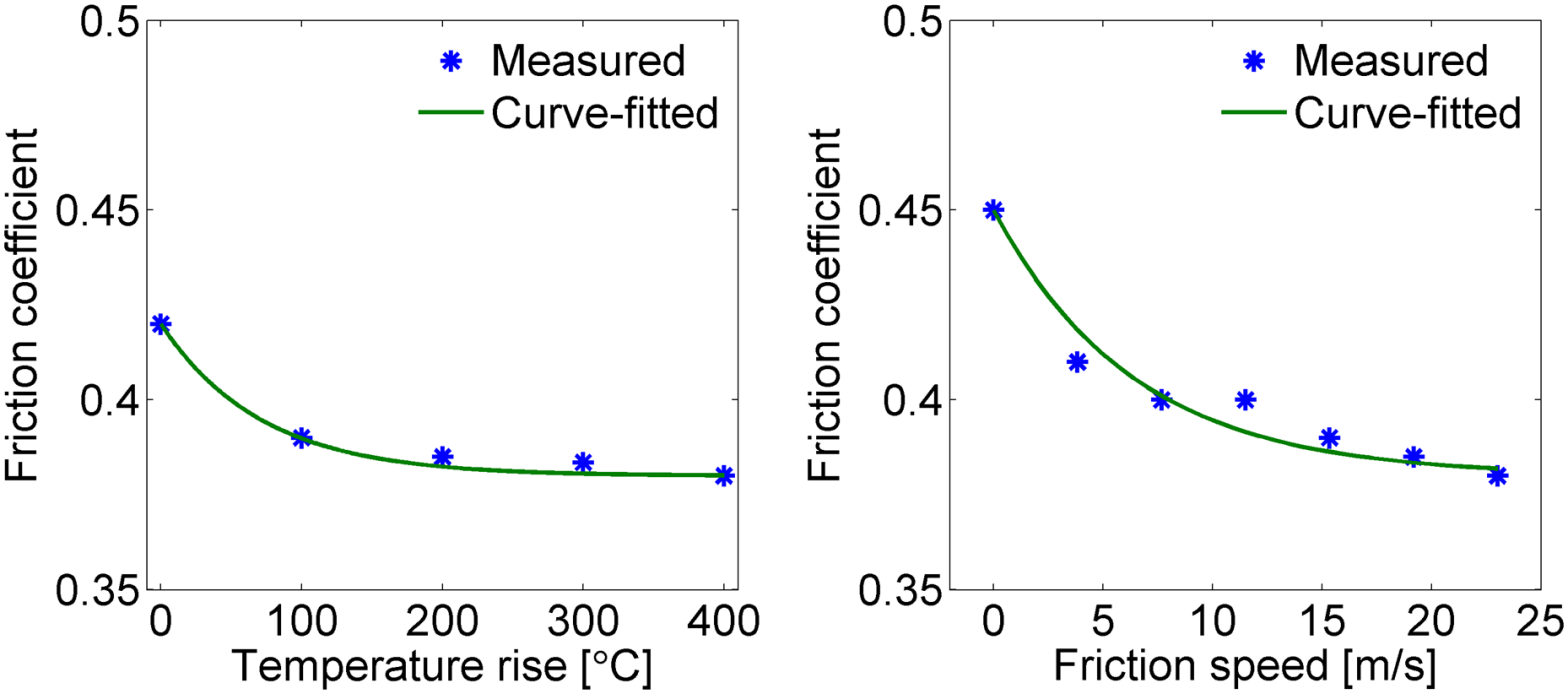
Measured and curve-fitted friction coefficients between the brake disc and the pad. The left portion of the figure shows the variation of the friction coefficient due to the temperature change at a friction speed of 9.6 m/s, and the right portion of the figure shows the variation of the friction coefficient due to the friction speed change at a temperature of 50°C.

Eq (2) is derived based on the dynamometer test results for a specific disc and pad. Fig 2 shows the dynamometer test results for variable friction coefficients between the disc and the pad due to the increase in temperature and friction speed. The asterisk points imply measured values, and the solid lines imply curve-fitted values using Eq (2) and the parameter values in Table 1. Fig 2(a) shows that the friction coefficient between the disc and the pad decreases as the surface temperature of the disc and the pad increases at a friction speed of 9.6 m/s. The friction speed is the tangential speed of the contact point of the disc and the pad. Fig 2(b) shows that the friction coefficient between the disc and the pad decreases as the friction speed increases at a surface temperature of 50°C. By comparing the measured and curve-fitted values in Fig 2, we see that the proposed equation (Eq (2)) validly represents the varying characteristics of the brake friction behaviors. Friction speed 20 m/s in Fig 2 corresponds to 113 km/h vehicle speed for the present HILS system since the wheel diameter is 0.86 m and the wheel disc diameter is 0.55 m for the present HILS system.

**Table 1:** Parameter Values for the Variable Friction Coefficients of a Railway Vehicle Considered in This Paper.

Parameter	Value
*n*_*v*_	0.184
*m*_*v*_	0.1
*n*_*T*_	0.105
*m*_*T*_	0.014
*μ*_*d0*_	0.38

Table 1 shows the parameter values of Eq (2) for the variable friction coefficients of the railway vehicle considered in this paper; these parameters were obtained by dynamometer tests. The correction constant *c* is for matching equation values with test results, and it is 0.98 for the present case. The railway vehicle (intercity transit) is in service commercially in Korea with a 150 km/h normal speed and has wheel-mounted disc brakes. The disc was made of steel alloy and the pad was made of organic materials, such as rubber-resin bonded with metal fibers and some additives.

To estimate the disc temperature increase *T*_*d*_ in Eq (2), a simple energy balance equation was considered for the brake system of the railway vehicle instead of full partial differential equations. The generated power on the disc is the product of the braking force and the braking speed, and the generated power reduces the train speed and is then primarily converted to thermal energy. There are two major types of heat dissipation: heat conduction to the axle on which discs are mounted, and heat convection to the air in which the rate depends on the vehicle’s speed. The energy balance equations used in this paper are as follows.

$${\dot{E}}_{d}(t)={\dot{E}}_{br}(t)-({\dot{Q}}_{cond}(t)+{\dot{Q}}_{conv}(t))$$ (3)

$${\dot{E}}_{br}(t)=\sigma_{d}\;T_{b}(t)\;\omega_{d}(t)$$ (4)

$$\begin{array}{ll} {\dot{Q}}_{cond}(t)+{\dot{Q}}_{conv}(t) & =h_{d}\;A_{axle}\;T_{d}(t)+h_{v}(t)\;A_{disc}\;T_{d}(t)\\ & =\alpha \;T_{d}(t)+\beta \;T_{d}(t)\;v_{w}{\left(t\right)}^{0.8} \end{array}$$ (5)

$$T_{d}(t)=\frac{1}{c_{p}\;m_{disc}}\;\int_{0}^{t}{\dot{E}}_{d}(\tau )\;d\tau$$ (6)

where *E*_*d*_ is the energy stored in the brake disc, *E*_*br*_ is the energy generated by the brake friction force, *Q*_*cond*_ is the energy dissipated by conduction to the axle, *Q*_*conv*_ is the energy dissipated by convection to the air, *σ*_*d*_ is the ratio of the thermally converted power to the mechanical power in the disc, *ω*_*d*_ is the angular speed of the disc, *h*_*d*_ is the thermal conductivity from the disc to the axle, *h*_*v*_ is the convection coefficient, *A*_*axle*_ is the area of conduction, *A*_*disc*_ is the area of the disc’s convective surface, *c*_*p*_ is the specific heat of the disc at a constant pressure, *m*_*disc*_ is the mass of the disc, *v*_*w*_ is wheel (circumferential) speed, and *α* and *β* are two equivalent constants.

In Eq (5), the convection coefficient *h*_*v*_ is approximated by the value that is proportional to (wheel speed)^0.8^ according to the result from reference [23,24], and then Eq (5) is expressed using constants *α* and *β*. The two values of *α* and *β* are determined so that dynamometer test results are approximately the same as the simulation results for the disc’s rate of the temperature increase and the maximum temperature.

## Modeling

**Fig 3:**
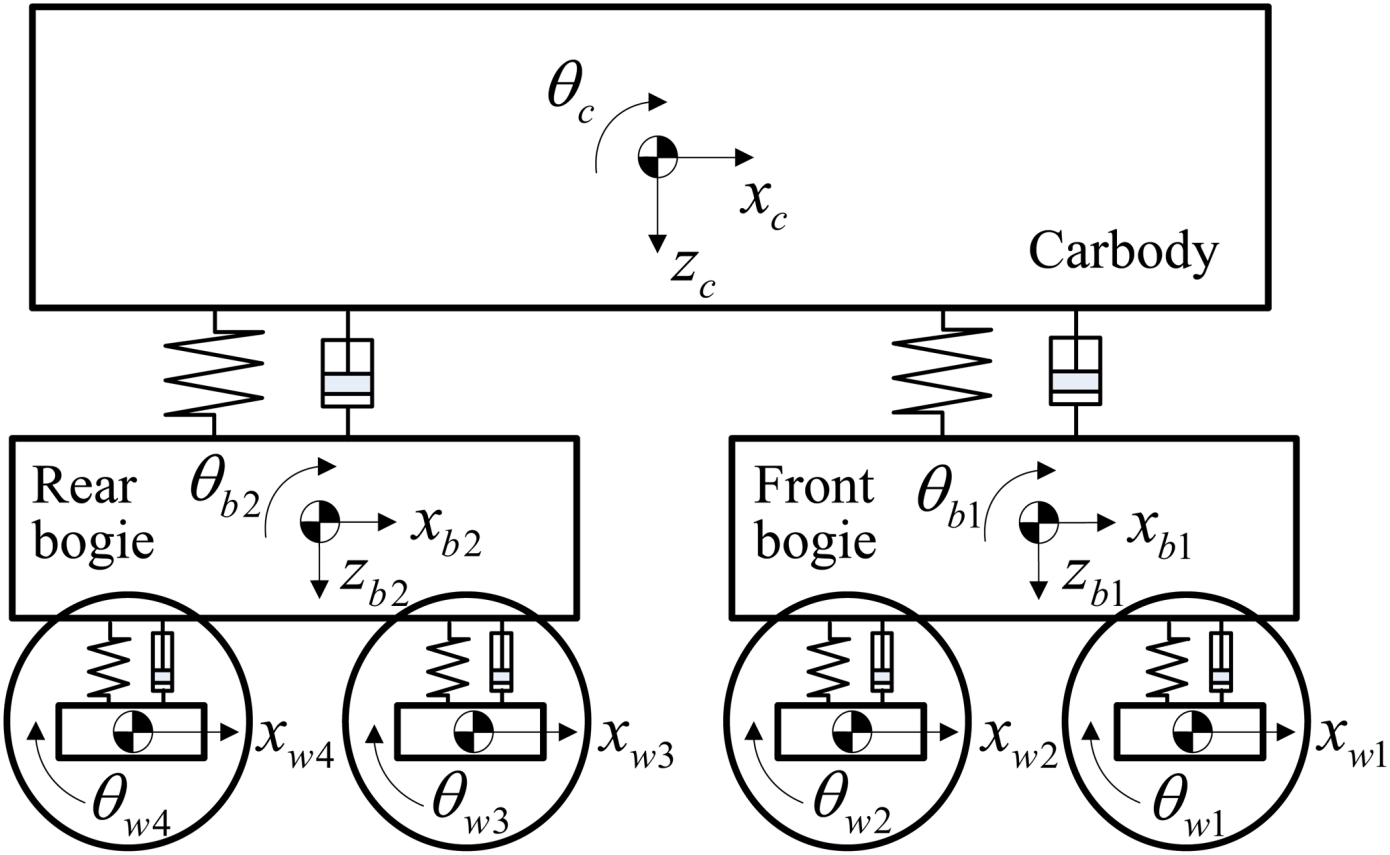
Schematic of the vehicle dynamic model. We assume the train runs on rails arranged in a straight line without any curves; thus, each mass is modeled as having plane motion with 3 DOF, i.e., longitudinal, vertical and pitch motions. Moreover, we assume that the wheels are always in contact with the rails so that the wheelset motions can be represented by only two independent variables: *x* and *θ*. Therefore, a vehicle including a carbody, two bogies and four wheelsets is represented by 17 motion DOF.

For simulation purposes, we consider one vehicle of the target train with one carbody, two bogies and four wheelsets, as shown in Fig 3. We assume the train runs rails arranged in a straight line without any curves because lateral and spinning motions between the wheels and rails do not influence the drive dynamics significantly. Therefore, each mass can be modeled as having a plane motion with 3 DOF (degrees of freedom), i.e., longitudinal, vertical and pitch motions. Moreover, we assume that the wheels are always contacted to the rails so that the wheelset motions can be represented by only two independent variables: *x* and *θ*. Then, because the vehicle is composed of one carbody, two bogies and four wheelsets, its motion has a total of 17 DOF. Primary suspensions are installed between the bogies and the wheelsets, and secondary suspensions are installed between the carbody and the bogies. The subscripts, *c*, *b*, and *w*, refer to the carbody, bogie and wheelset, respectively, and subscripts 1, 2, 3, and 4 refer to the ordering of the location of the wheelsets in the vehicle.

**Fig 4:**
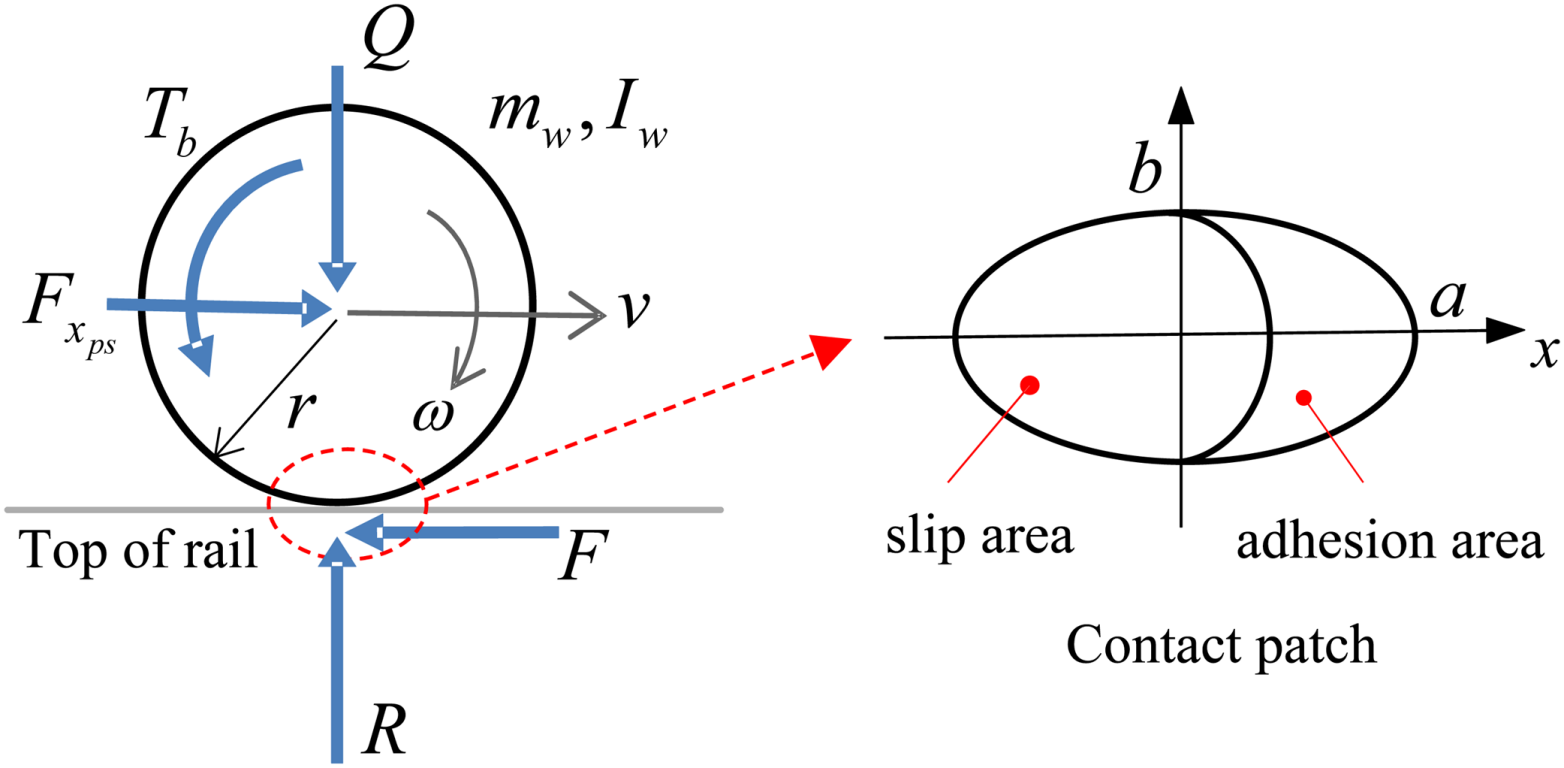
Free-body diagram of a wheelset and the shape of the contact patch. The wheel-rail contact is modeled by an elastic rolling contact in which the leading part of the oval contact patch is an adhesion area and the trailing part is a slip area. In the adhesion area, there is no slip velocity, but in the slip area, there is a relative velocity between the wheel and the rail.

Fig 4 shows a free-body diagram of the wheelset and the contact patch that has an oval shape, both of which are used in this simulation. The external forces acting on the wheelset are the vertical force *Q*, the longitudinal force *F*_*xps*_ due to the primary suspension, the normal reaction force *R* by the rail, the braking torque *T*_*b*_ and the adhesion force *F* due to the wheel-rail contact. Using the free-body diagram, two equations for the longitudinal and the rotational motions of the wheelset can be written as follows.

$$I_{w}\dot{\omega }(t)=F(t)\;r-T_{b}(t)$$ (7)

$$m_{w}\dot{v}(t)=F_{x_{ps}}(t)-F(t)$$ (8)

where *I*_*w*_ is the moment of inertia of the wheelset, *ω* is rotational speed of the wheelset, *v* is the linear velocity of the wheelset, *r* is the rolling radius of the wheel, and *m*_*w*_ is the mass of the wheelset.

The adhesion force *F* is generated by a complex tribological phenomena between the wheels and the rails, and this force significantly affects the performance of the drive dynamics. Previous studies have introduced a simplified contact model to easily compute the adhesion force, which can reduce the calculation time and represent wheel slide behaviors subject to large creep conditions on contaminated contact surfaces [16,17]. The simplified contact model for the adhesion force is applied for this study.

The simplified wheel-rail contact model for the adhesion force *F* is as follows [19].

$$F(t)=\frac{2\;\mu (t)\;Q(t)}{\pi }\left(\frac{k_{A}\;\varepsilon (t)}{1+{\left(k_{A}\;\varepsilon (t)\right)}^{2}}+\text{arctan}\left(k_{s}\;\varepsilon (t)\right)\right)$$ (9)

with

$$\varepsilon (t)=\frac{2}{3}\;\frac{C\;\pi \;a^{2}\;b}{Q(t)\;\mu (t)}\;s(t)$$ (10)

$$\mu (t)=\mu_{0}\;\left[\left(1-A\right)\;e^{-B\;\left|v(t)-r\;\omega (t)\right|}+A\right]$$ (11)

$$s(t)=\frac{v(t)-r\;\omega (t)}{v(t)}$$ (12)

where *Q* is wheel load, *μ* is the friction coefficient between the wheels and the rails, *k*_*A*_ is the reduction factor in the adhesion area, *k*_*s*_ is the reduction factor in the slip area, *μ*_*0*_ is the maximum friction coefficient at zero slip velocity, *A* is the ratio of the friction coefficient at the infinity slip velocity to the maximum friction coefficient at the zero slip velocity *μ*_∞/_ *μ*_*0*_, *B* is the coefficient of the exponential friction decrease, *C* is the proportionality coefficient characterizing the contact shear stiffness, and *a* and *b* are the half-axes of the contact ellipse, and *s* is total creepage.

In this contact model, we assume that the contact area is composed of the adhesion area and the slip area, that the surface shear stress on the slip area is proportional to the surface normal stress obtained using the Hertz theory of elastic contact [25], and that the surface shear stress on the adhesion area increases linearly with the slope *C*, which is derived using Kalker’s linear theory [26].

In the contact model, one parameter set *A*, *B*, *k*_*A*_, *ks* and *μ*_*0*_ is introduced to define the condition of the wheel-rail contact and the diverse combination of the parameters can represent various wheel-rail contact situations, such as dry, wet or contaminated conditions.

Parameter values for the wheel-rail contact model of the target vehicle are shown in Table 2. The parameter values are slightly modified from the recommended values in reference [16] for wet conditions to emphasize the effect of a variable friction coefficient between the disc and the pad.

**Table 2:** Parameter Values for a Wet Condition of a Wheel-Rail Contact Model of the Target Vehicle.

Parameter	Value
*k*_*A*_	0.30
*ks*	0.10
*μ*_*0*_	0.21
*A*	0.40
*B*	0.20

## The Brake HILS System

**Fig 5:**
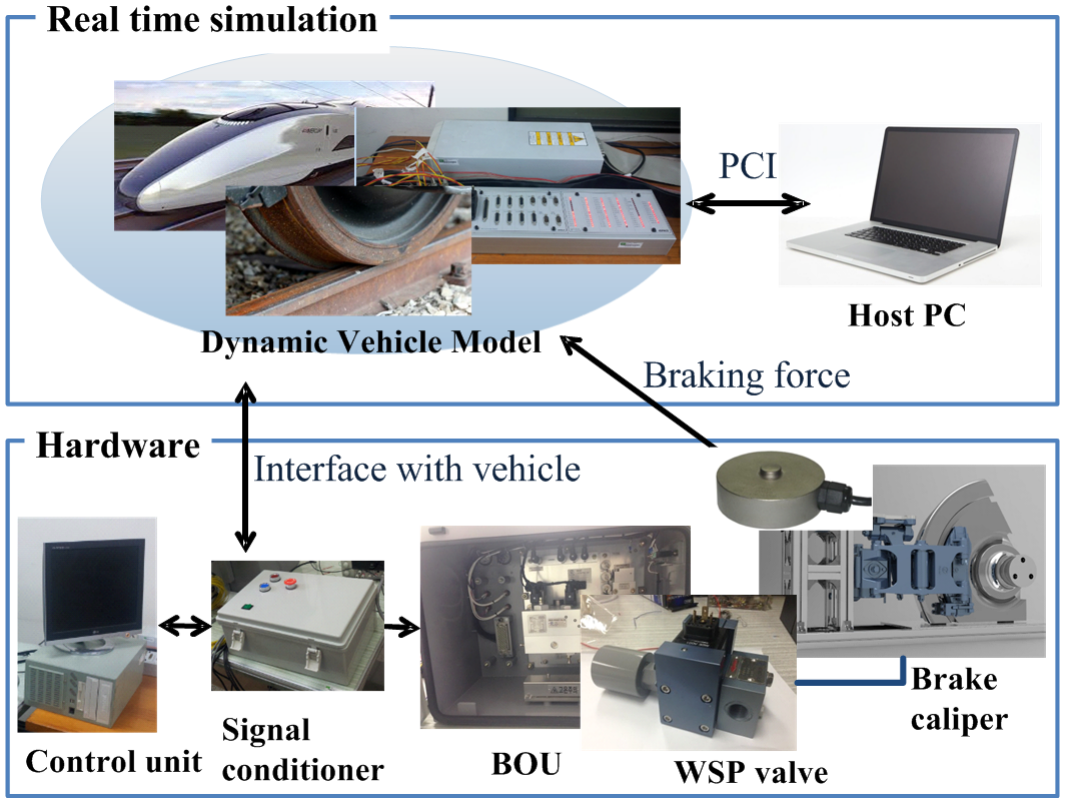
Schematic configuration of the brake HILS system. Vehicle dynamics were simulated in real-time using a high-speed dSPACE unit, and the four wheel speeds simulated by the dSPACE unit interfaced with the actual brake hardware through the signal conditioner and the BOU. The braking force measured by the load cells was delivered as feedback to the dSPACE unit to compute the wheel speeds at the next sampling instant.

A hardware-in-the-loop simulation (HILS) system for a brake unit of a railway vehicle considered in this research is composed of the dynamic model of the railway vehicle for real-time simulations and of the hardware components of the brake system, as shown in Fig 5. The dynamic model of the real-time simulations consists of the vehicle dynamics, the wheel-rail contact model, and the varying friction model of the disc and the pad, as described in previous section. The hardware components of the brake system include the brake caliper, the brake operating unit (BOU), four WSP (wheel slide protection) valves, four reservoirs for dummy volumes, the brake force sensors, the air pressure sensors, the signal conditioners, the brake controller, and the pneumatic pipes. The brake controller is composed of an ECU (electronic control unit) and an ASCU (anti-skid control unit) whose functions are brake force control using brake control and wheel slip prevention logic. The signal conditioners amplify and filter the ECU and the ASCU output signals and send the signals to the BOU. The BOU actuates pneumatic valves and then actuates the brake caliper. The piping work was performed as closely as possible to the pneumatic system of the actual service train (target train). The mechanical brake device in the developed HILS system has the same structure with the schematics in Fig 1, but the wheel is stationary. The motion of the vehicle is implemented by the real-time simulation part in the HILS. The HILS system is able to provide actual normal brake force on the brake disc. Then the friction force on the brake disc is calculated by the product of the measured normal force and the friction coefficient of the brake pad.

Co-simulation using the dynamic models and the hardware components requires proper data exchange between the hardware components and the software vehicle models. The software vehicle models provide the simulated vehicle speeds to the hardware components, and then the BOU generates the braking force using the brake caliper according to the brake command and the simulated vehicle speeds. The braking force was measured by the brake force sensor (load cell), and the measured braking force was fed back to the software vehicle model. Therefore, from the viewpoint of the hardware brake unit, the brake unit of the brake HILS system receives similar electrical signals with signals of the actual train. The brake unit actually does not know whether the electrical signals come from the actual train or from the HILS system. In other words, we can test the brake unit using the HILS system instead of the actual train.

**Fig 6:**
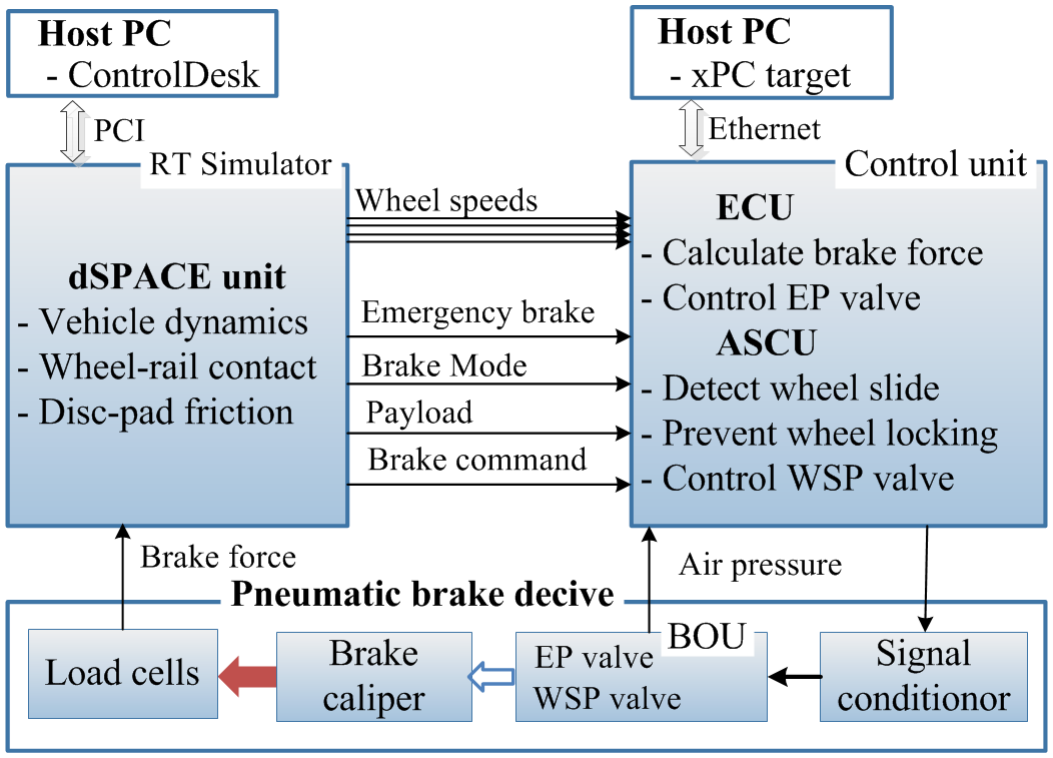
The signal flow and interfacing of the brake HILS system. There are two host computers: one computer is for the dSPACE unit in which the vehicle dynamics are simulated in real-time, and the other computer is for the control unit in which brake force is calculated and the wheel slide is detected and protected. The braking force is generated by the pneumatic brake device according to the output signals of the control unit.

The signal flows in the HILS system are shown in Fig 6. The host PCs generate a brake command and monitor the simulated train-running status using the man-machine interface (MMI) programmed by using the ControlDesk software. Railway vehicle dynamics and wheel-rail contact/pad-disk contact mechanics run in real-time using a high-performance DSP board, and vehicle speeds and wheel slide are generated as real-time simulation results. Then, the generated four wheelset speeds are sent to the control unit, i.e., the ECU and the ASCU. The control unit and the signal conditioners make control signals that are converted to power signals to actuate pneumatic valves. The braking force generated by the BOU and the brake caliper was measured by the load cells and was sent to the vehicle dynamics as an input.

The ECU controls an electro-pneumatic (EP) valve that generates the required pressure using a PI (proportional-plus-integral) control and a hysteresis-compensating algorithm to achieve the required brake effects corresponding to commands from the GUI. The ASCU control logic is used to detect skid and to prevent the wheels from locking using the optimal adhesion force.

**Fig 7:**
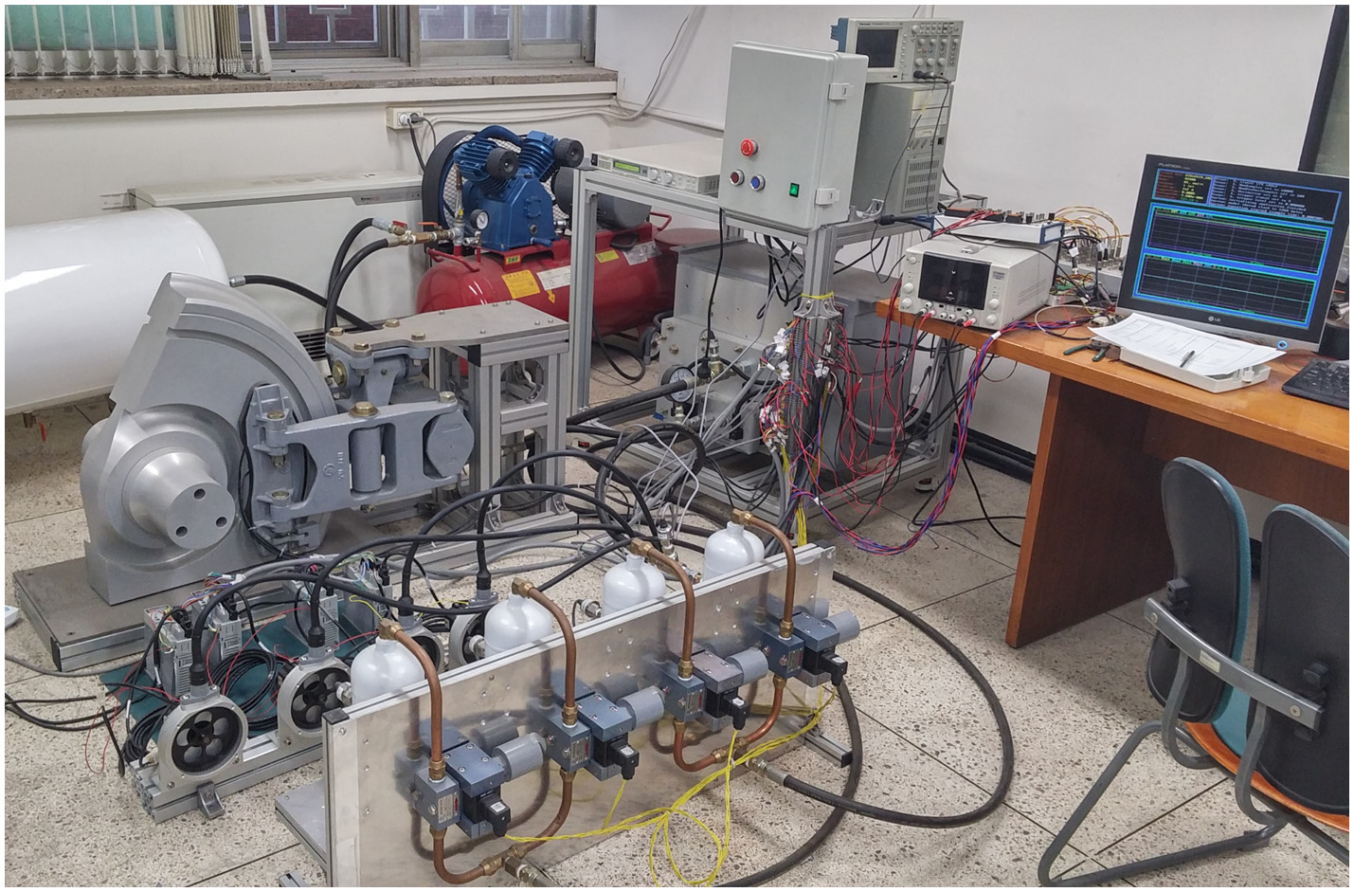
Picture of the brake HILS system developed in our laboratory. The red device on the upper side of the picture is a portable air compressor used to deliver power to the mechanical brake unit. The wheel and the brake caliper are installed on the floor (the center of the picture), the BOU and the dSPACE unit are placed on the table (the right side of the picture), and four WSP valves and four white reservoirs corresponding to piping and cylinder volumes are placed on the vertical panel (the bottom of the picture).

The hardware of the pneumatic brake unit for the HILS is prepared using a commercial product from the target vehicle except for the electronic controller. The electronic controller of the HILS system is achieved using the MATLAB/Simulink software, the xPC target board, and a desktop PC to implement the control logic described in the previous section. The vehicle model, as well as the wheel-rail contact model, is simulated in real-time using a 0.1 ms sampling time by means of a high-performance dSPACE unit. Additionally, the signal conditioners are prepared to interface the control signals with the actuator signals. Fig 7 shows a picture of the brake HILS system developed in our laboratory.

## Results and Discussion

To run the brake HILS system, a braking scenario was prepared as follows. One intercity vehicle that runs at a service speed of 150 km/h on a straight rail without irregularities was used, and the rail was operated in wet conditions. The parameters of the wheel-rail contact model in Table 2 were used. The EB (emergency brake) command was ordered by the driver at the service speed of 150 km/h. The deceleration value of the emergency braking was set to 5 km/h/s. All the other parameters have values of the target railway vehicle, as shown in Table 3.

**Table 3:** Target Vehicle Parameter Values. COG implies the center of gravity.

Parameters	Values
Mass of a carbody	35,000 kg
Mass of a bogie	2,500 kg
Mass of a wheelset	850 kg
Moment of inertia of a carbody, *I*_*yy*_	1,200,000 kg·m^2^
Moment of inertia of a bogie, *I*_*yy*_	950 kg·m^2^
Moment of inertia of a wheelset, *I*_*yy*_	280 kg·m^2^
Height of COG of a carbody	1.2 m
Height of COG of a bogie	0.62 m
Height of COG of a wheelset	0.43 m
Bogie base	14 m
Wheel base	2.5 m
Vertical stiffness of a secondary spring	700 kN/m
Longitudinal stiffness of a secondary spring	200 kN/m
Vertical stiffness of a primary spring	2,000 kN/m
Longitudinal stiffness of a primary spring	18,000 kN/m
Stiffness of traction link	13,000 kN/m

**Fig 8:**
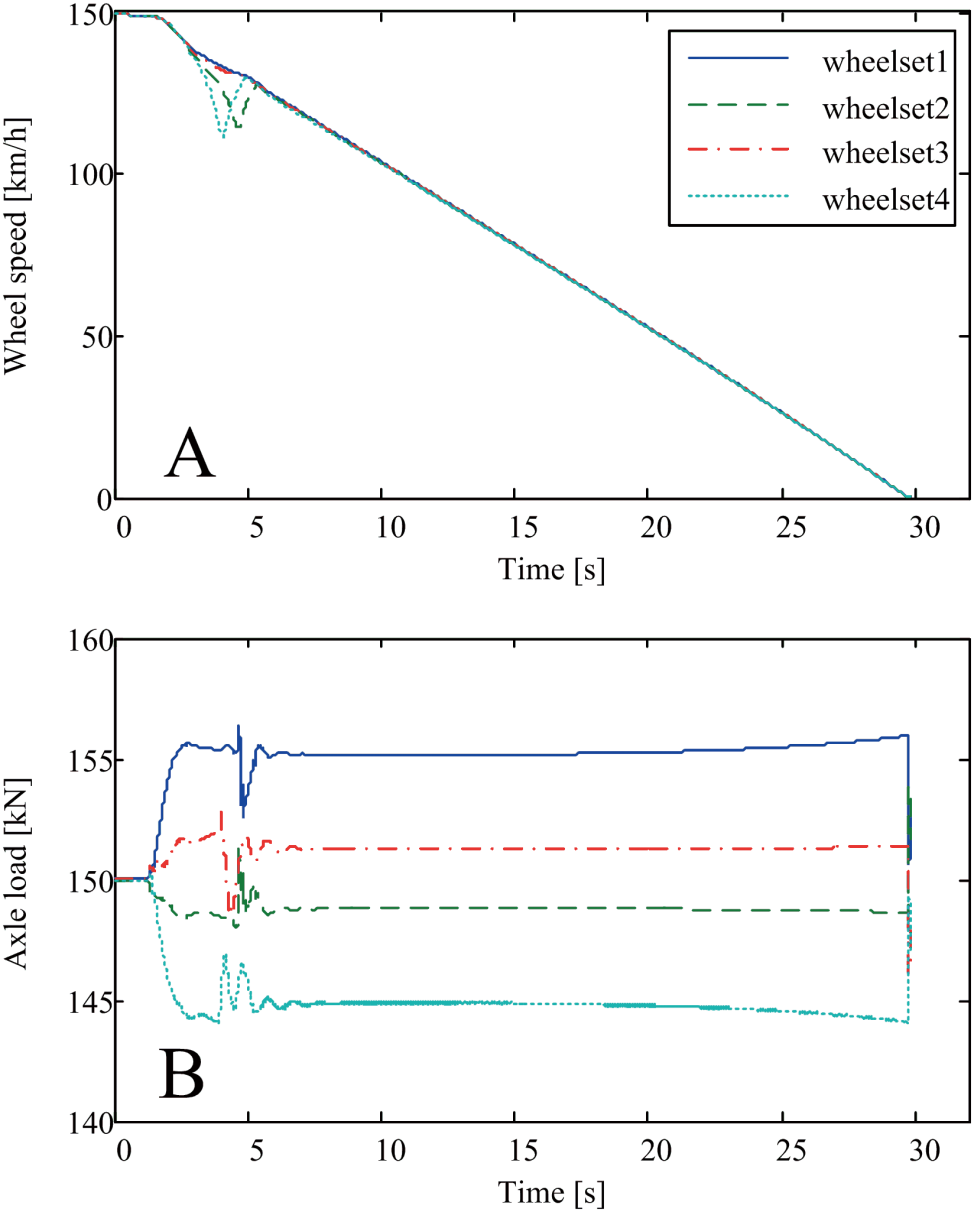
HILS system experimental results for various wheel speeds and axle loads. (A) The circumferential speeds of four wheelsets during emergency braking. (B) The axle loads of four wheelsets during emergency braking.

Fig 8 shows the experimental results for the four wheel speeds and the four axle loads during emergency braking of the HILS system. These HILS results show that it takes approximately 30 s to stop the train completely when the EB command is given at a train speed of 150 km/h. The deceleration and braking forces in Fig 8 that were generated by the HILS system are acceptable in view of the specified braking performance of the target train. There are particular braking behaviors at approximately 4 seconds, at which time wheel slide occurs in the second and fourth wheelsets and the WSP valves begin to operate. The reason wheel slides were observed in only the second and fourth wheelsets is that the required adhesion coefficient limit of the wheel was increased at the second and fourth wheelsets because the axle load decreased due to the weight transfer during braking. In this HILS test, we used the variable friction coefficient *μ*_*d*_ between the disc and the pad instead of the constant friction coefficient *μ*_d0_.

**Fig 9:**
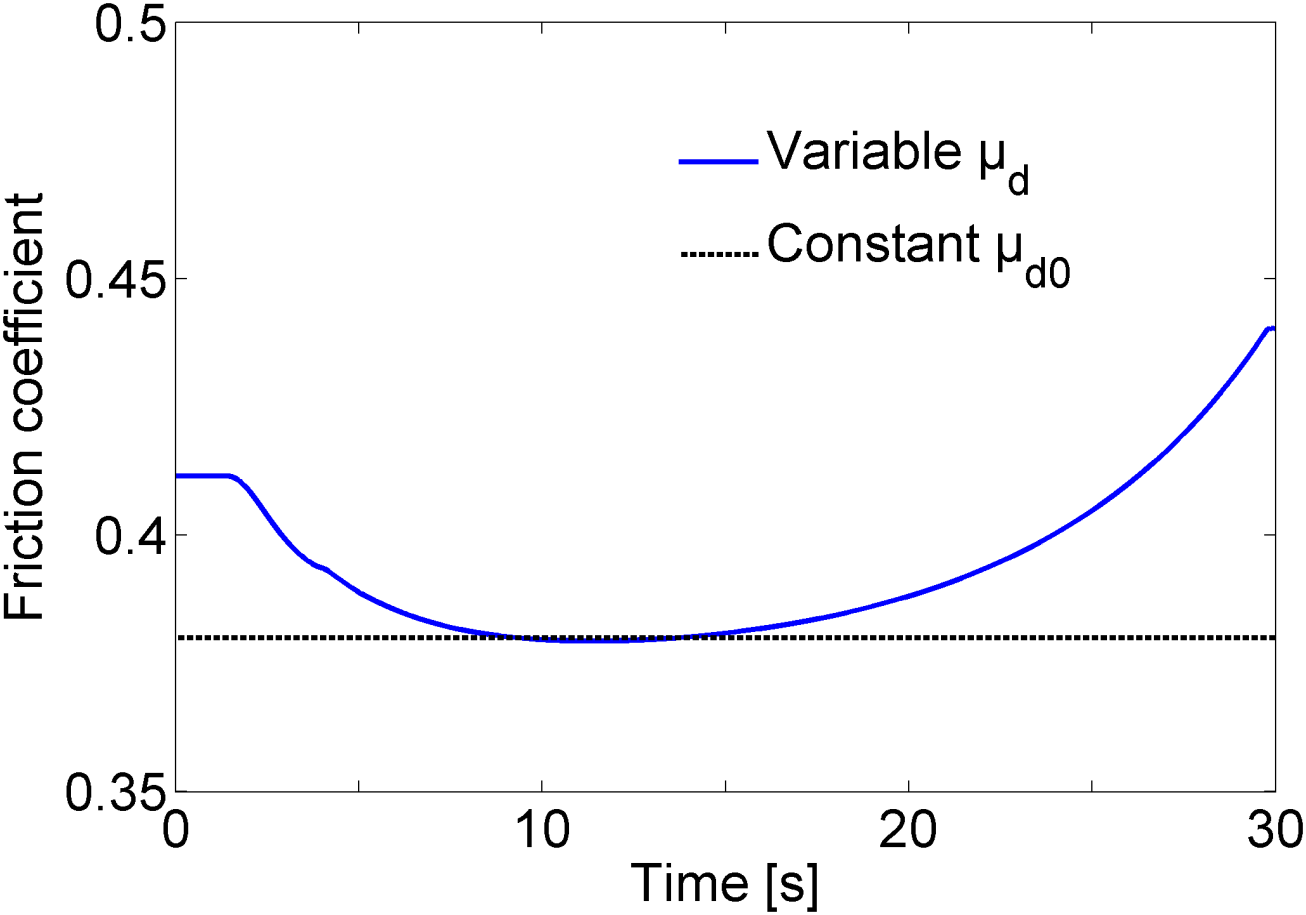
Friction coefficients between the disc and pad during emergency braking of the HILS system. The variable friction coefficient at the initial and final stage of braking is greater than the friction coefficient at the intermediate stage due to the low temperature of the brake materials and due to the low friction speed.

The variable friction coefficient between the disc and the pad used for emergency braking of the HILS system is shown as a solid line in Fig 9. This curve implies that the friction coefficient is high at the initial stage of braking due to the low temperature of the brake materials, and that it is high at the final stage of braking due to the low friction speed compared with the friction coefficient of the intermediate stage of braking. As predicted by Eq (2), the variable friction coefficient *μ*_*d*_ was greater than the constant friction coefficient *μ*_d0_, which was 0.38 for the present case. Even though the experimental results of the dynamometer in Fig 2 cannot be compared directly with the predicted values in the solid line of Fig 9 because of different abscissa, the decrease of the friction coefficient at the initial stage of braking and the increase of the friction coefficient at the final stage of braking in Fig 9 demonstrate partially the validity of the proposed Eq (2).

**Fig 10:**
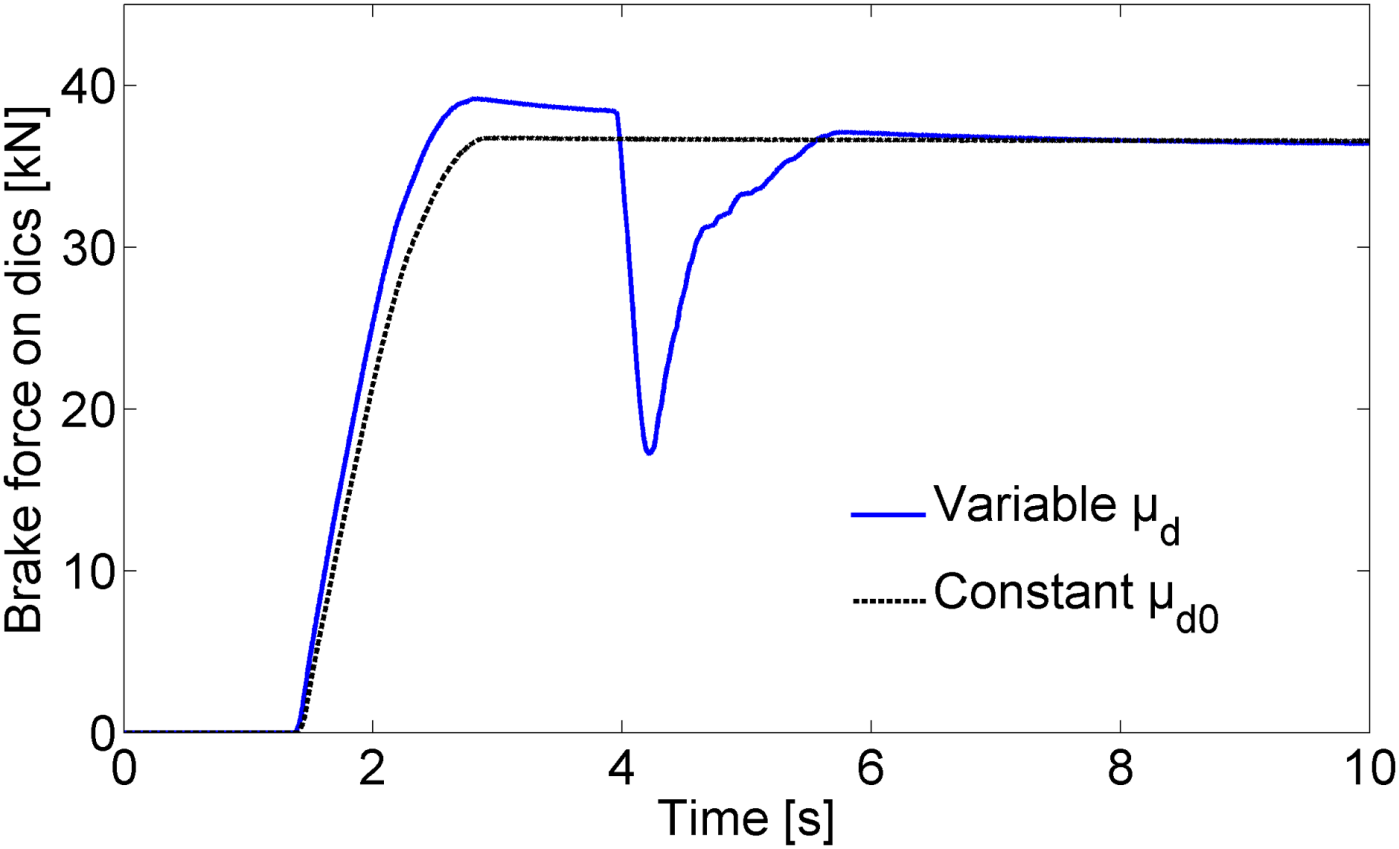
HILS results for the tangential brake forces on the disc during emergency braking. The depression of the blue solid line at approximately 4 s is due to releasing the brake force for the readhesion from the wheel slide by opening the WSP valve of the sliding wheelset. The gree dotted line corresponds to the braking force using a constant friction coefficient in which no wheel slide is simulated during braking even if wheel slide actually occurs.

**Fig 11:**
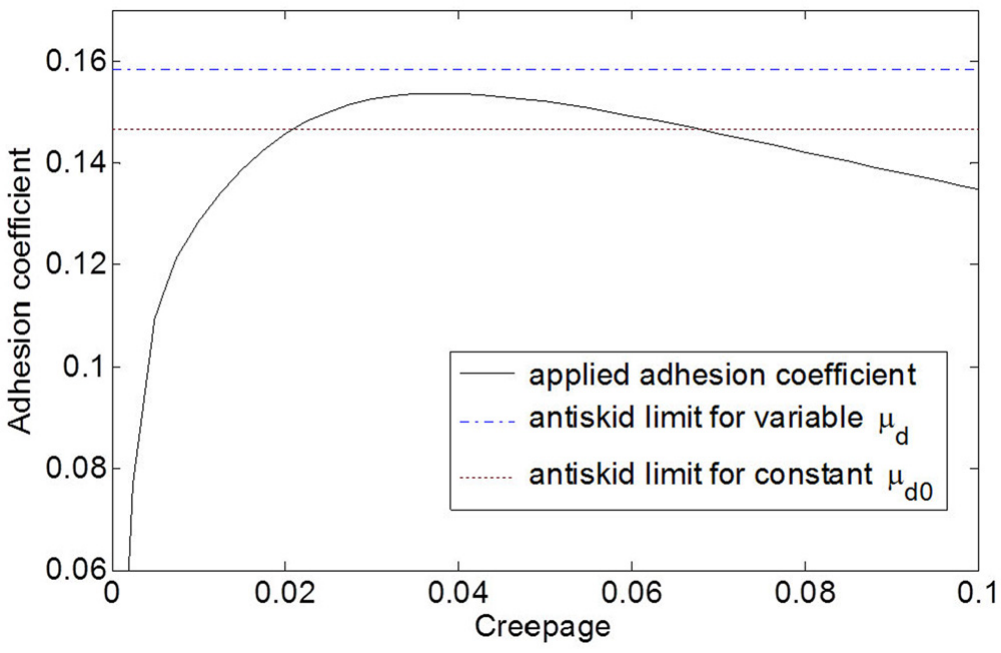
Adhesion coefficient as a function of creepage. Greater brake force due to the variable friction coefficient requires greater adhesion coefficient in the wheel-rail contact for no wheel slide, but the achieved adhesion coefficient is less than the required limit value as shown in the blue dash-dot line, and thus, wheel slide occurs.

Interestingly, if we use the constant friction coefficient *μ*_d0_ instead of the variable friction coefficient *μ*_*d*_ for the same braking scenario, as shown in Fig 8, no wheel slide, even in the second and fourth wheelsets, occurs during the HILS test. The solid line in Fig 10 shows the tangential brake force on the disc of the fourth wheelset generated by the variable friction coefficient whereas the dotted line in Fig 10 shows the tangential brake force generated by the constant friction coefficient *μ*_d0_. The greater the brake force generated by the variable friction coefficient, the greater adhesion coefficient required in the wheel-rail contact to prevent wheels from sliding. The achieved maximum adhesion coefficient between a wheel and rail was less than the required limit value for variable *μ*_*d*_, and bigger than the required limit for constant *μ*_d0_, as shown in Fig 11. Therefore, wheel slide only occurs for the case with the variable friction coefficient, as shown in the solid line of Fig 10. When wheel slide occurs in a specific wheelset, the brake force of that wheelset decreases to release the wheel slide by opening the WSP valve of that wheelset, as observed in the solid line in Fig 10. The dotted line in Fig 10 shows that constant brake force without any potholes results in no wheel slide during braking. Note that the applied normal force on the brake disc of the fourth wheelset was directly measured using three load cells installed between the brake caliper and the wheel, while the applied normal forces of the other three wheelsets were calculated from measured pressures of reservoirs corresponding to brake cylinders in the present HILS system as shown in Fig 7.

In view of wheel slide phenomena of a wheelset, the most important thing is whether the applied brake force is greater than the available maximum adhesion force. Fig 11 shows the adhesion coefficient as a function of the creepage in the wheel-rail contact at approximately three seconds after the EB command (i.e., at an approximately speed of 140 km/h). The adhesion coefficient was computed from Eq (9) using

$$\text{Adhesion coefficient }=\frac{F}{Q}$$ (13)

To compute adhesion coefficient in Fig 11, we have used Eqs (9)–(12) and the parameter values in Table 2.

Furthermore, Fig 11 shows the two anti-skid limits required for the cases with the variable friction coefficient and the constant friction coefficient. In Fig 11, the anti-skid limit for the variable friction coefficient (dash-dot line) exceeds the maximum value of the adhesion coefficient (solid line), which means that the brake force generated on the disc exceeds the maximum adhesion force sustainable on the rail, and this force generates wheel slide. Conversely, the anti-skid limit for the constant friction coefficient (dotted line) is below the maximum value of the adhesion coefficient (solid line), which means the brake force generated on the disc is less than the maximum adhesion force sustainable on the rail, and then, there is no wheel slide with roughly constant deceleration.

If the achieved brake force with the variable friction coefficient exceeds the maximum adhesion force, this triggers the wheel slide and the creepage of the wheel increases. Increased creepage of the wheel reduces the adhesion force. Then, the wheel slide protection (WSP) logic of the ASCU detects the wheel slide and activates the WSP valve to recover the wheel speed. In Fig 10, we see the WSP valve starts at approximately 4 s. Although the two cases with the variable friction coefficient and the constant friction coefficient show similar behaviors at the beginning stage of braking, the case with the variable friction coefficient generates wheel slide, but the case with the constant friction coefficient does not generate wheel slide for the present braking scenario of the HILS system. Therefore, for more realistic brake simulations that represent actual braking conditions of the train, we should take the varying friction coefficients between the disc and the pad into account. If we consider only a constant friction coefficient instead of the variable friction coefficient, we can miss some critical phenomena, such as the occasional wheel slide, as shown in Fig 10.

**Fig 12:**
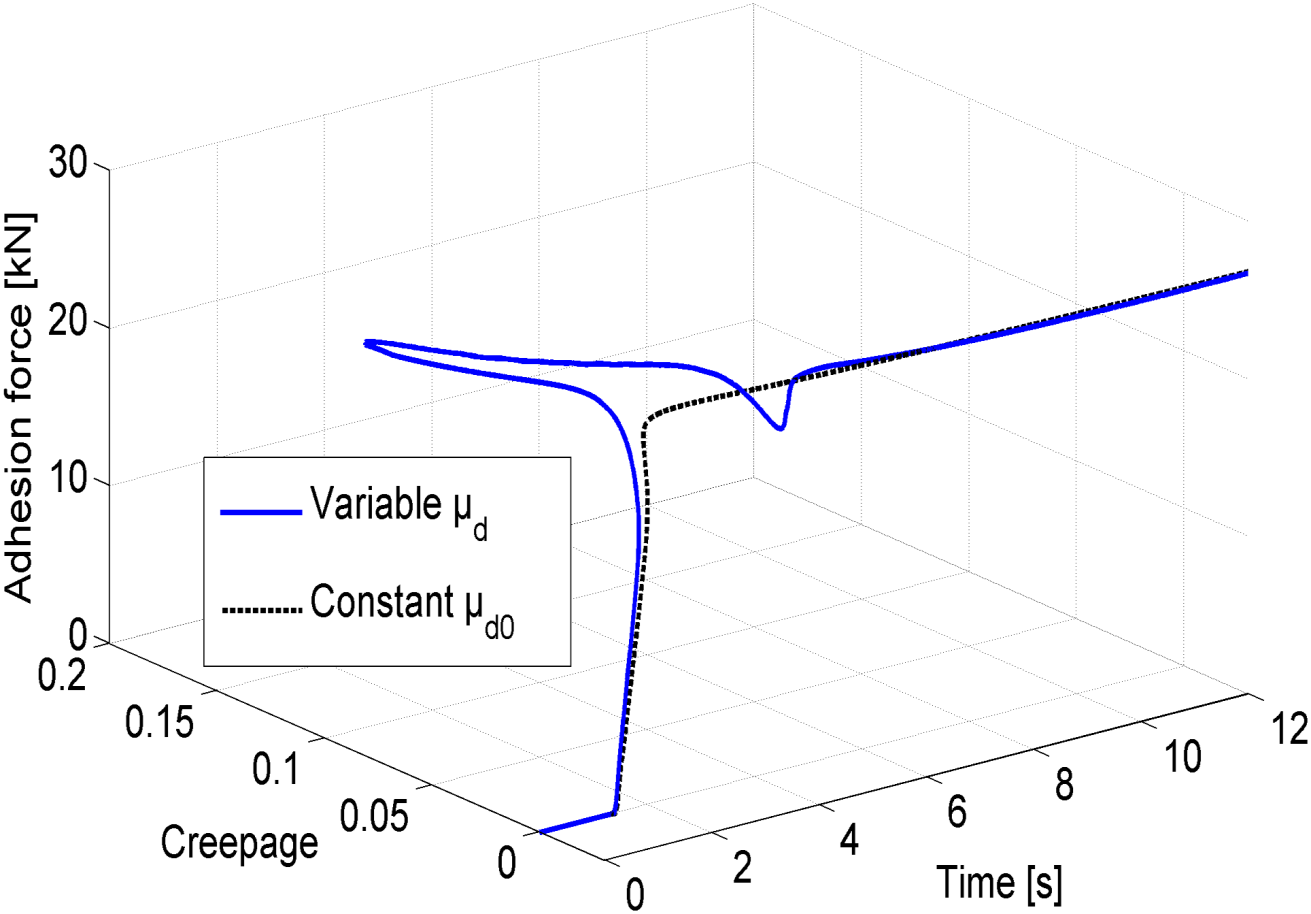
Experimental results of the HILS system for the adhesion forces. The blue solid line and the green dotted line correspond to the adhesion force as functions of the creepage and of time for the case using the variable friction coefficient and the constant friction coefficient, respectively.

Fig 12 shows the adhesion forces of the fourth wheelset as functions of the creepage and of time for the cases with variable and constant friction coefficients in a three-dimensional plot. Although the above phenomenal difference for the variable and constant friction coefficient cases does not occur frequently during brake operations, the variable friction coefficient between the disc and the pad is able to bear a better resemblance to the actual train braking process, especially in the beginning and final stages of braking.

## Conclusions

For a mechanical brake system of a railway vehicle, the effect of a varying friction coefficient between the disc and the pad has been analyzed and demonstrated in this paper using a brake HILS system developed in our laboratory. The effect of the varying friction coefficient between the disc and the pad occasionally appeared in a significant manner at the beginning and final stages of braking.

A formula for the variable friction coefficient between the disc and the pad was introduced based on measured data from dynamometer tests in which the variable friction coefficient using five parameters is a function of the friction speed and the increase in the temperature. Then, the formula for the variable friction coefficient was applied to the emergency braking in a brake HILS system in which we installed the actual brake unit hardware of a 150 km/h intercity transit train. The validation for the formula was done with one comparison with results of dynamometer test. Even though the simplified formula with 5 parameters was not sufficient to achieve detail phenomena of tribology between the brake disc and the pads, it has the compromised complexity to simulate and to estimate the behavior of the wheel sliding in brake device in a HILS system.

The validity of the variable friction coefficient of a disc pad has been demonstrated by HILS tests for an emergency braking scenario with an initial velocity of 150 km/h on a wet rail. HILS results showed that the formula for the variable friction coefficient clearly represents the physical phenomena such that the variable friction coefficient at the initial and final stage of braking is greater than the friction coefficient at the intermediate stage due to the low temperature of the brake materials and due to the low friction speed. The HILS test demonstrated that the brake force when using the variable friction coefficient generated wheel slide but that the brake force when using the constant friction coefficient did not. Conclusively, the variable friction coefficient between the disc and the pad bears a better resemblance to the actual train braking process, especially in the beginning and final stages of braking.

Wheel slide is one of the critical considerations for braking performance, and wheel slide can be achieved more accurately in a brake HILS system when using a variable friction coefficient than when using a conventional constant friction coefficient.
